# Redemption

**Published:** 1890-06-21

**Authors:** 


### Words of Consolation.

REDEMPTION.

You have lost something. Your birthright, the inheritance which once belonged to your fathers, the home which should have been yours by right. One of your ancestors was cheated of it by a foul lie. Originally they had received it as a free gift from One who knew how to give all things richly, but this ancestor was ungrateful, and did not know the true value of the treasure. She listened to the deceiver who whispered evil thoughts about the Giver, and who offered to show her the way to get a much better estate than the one she was then enjoying. In an evil moment she took the cruel advice, and by one act forfeited the glorious future prepared for the whole human race. You have guessed, possibly, that she who lost your inheritance was Eve, and that the tempter who led her into sin was Satan. That wicked spirit offered to our first parent the enticing fruit. She saw that it was pleasant to the eye and good for food, and a tree to be desired to make one wise, but no sooner had she eaten than the punishment overtook her. She and Adam, the partner of her guilt, were driven from the garden of Eden for their disobedience, to gain their bread by the sweat of their brow.

With the curse, however, God gave a promise of redemption; the inheritance should some day be bought back again, and man be restored to his first estate. But who could hope to pay the tremendous price required? No created being, either man or angel, could be of any avail, so for centuries man drifted farther and farther away from his Creator, till the Almighty Himself condescended to be the Saviour. The God Man could alone by His death satisfy the justice of the Most High.

"There was no other good enough
To pay the price of sin,
He only could unlock the gates
Of Heaven and take us in."

But man had to be drawn by love and driven by fear ere he could be brought to see the necessity of a Saviour. The Almighty, in mercy and love, chose a people who might be taught the awful wickedness of sin and be shown the way back to Paradise. The law given to Moses on Mount Sinai was the schoolmaster to bring men to Christ. The whole teaching of the Old Testament points to redemption by the death of Christ. Without shedding of blood there could be no remission of sins, so the daily sacrifice of animals became a type of the one great atonement. A type only, for the blood of bulls and of goats could never take away sin, therefore in fullness of time, when men's minds had realized this, and they were longing for the promised Christ who, as man for man, should take our iniquities upon him, came the Saviour into the world. Once and for ever, our Jesus satisfied the eternal justice. Throughout His whole life—at the Last Supper and upon the bitter cross—Christ offered Himself for you, for me, for the sins of the whole world. Oh! full and perfect sacrifice, "which on the tender cross told the long hours of death, as one by one the pulse strings of that heart gave way."

Shall we be careless and ungrateful, thinking that because all this happened more than 1800 years ago we have no part or lot in this redemption now? The inheritance was given to Adam and Eve for all their descendants to enjoy, and the price paid bought it back for us and for all unto the end of time. Only we must not shrink from the responsibilities which our position brings with it. As God's chosen people we must believe, fear, and love the Father who made us, the Son who has redeemed us, the Holy Ghost who sanctifies us, and with thankful hearts receive the means of grace. Christ has left a memorial of His death and passion in the great sacrament of His love. Let us lift up our hearts to the glorious Godhead who planned and carried out our redemption.

